# High-level nitrogen removal achieved by Feammox-based autotrophic nitrogen conversion

**DOI:** 10.1016/j.wroa.2024.100292

**Published:** 2024-12-03

**Authors:** Xiaohui Cheng, Lanlan Hu, Tao Liu, Xiang Cheng, Jiyun Li, Kangning Xu, Min Zheng

**Affiliations:** a Beijing Key Lab for Source Control Technology of Water Pollution, College of Environmental Science and Engineering, Beijing Forestry University, Beijing, 100083, China; b Department of Civil and Environmental Engineering, The Hong Kong Polytechnic University, Hong Kong, 999077, China; c School of Environment, Tsinghua University, Beijing, 100084, China; d Water Research Centre, School of Civil and Environmental Engineering, University of New South Wales, Sydney, New South Wales, 2052, Australia

**Keywords:** Feammox, Anammox, Autotrophic nitrogen removal, Dissimilatory iron reduction bacteria

## Abstract

Anaerobic ammonium oxidation coupled with Fe(III) reduction (Feammox) is an essential process in the geochemical iron and nitrogen cycling. This study explores Feammox-based nitrogen removal in a continuous laboratory up-flow bioreactor stimulated by intermittently adding 5 mM Fe(OH)_3_ at intervals of approximately two months. The feed was synthetic wastewater with a relatively low ammonium concentration (∼100 mg N/L), yet without organic carbon in order to test its autotrophic nitrogen removal performance. The operation of this system showed the achievement of high-level ammonium and total nitrogen removal efficiency (∼97% and ∼90% on average, respectively) within four months of operation, along with a relatively practical rate of ∼50 mg N/(L·d). The demand of Fe(Ⅲ) for ammonium removal during the whole bioreactor operation was estimated to be only 0.033, two orders of magnitude less than that calculated based on the Feammox reaction producing nitrogen gas. A series of assays on Fe(II) oxidation with different oxidants (O_2_, NO_2_^−^ and NO_3_^−^) in abiotic and biotic batch tests further revealed an important role of Fe(II) oxidation processes, likely driven by microbial nitrate reduction and chemical oxygen reduction, in assisting the regeneration of Fe(III) for continuous Feammox-based nitrogen removal. This work demonstrates that Feammox-based autotrophic nitrogen conversion is a potential option for future wastewater treatment.

## Highlights

• Feammox-based autotrophic nitrogen conversion is studied in a continuous bioreactor.
• High removal efficiency for ammonium (>97%) and total nitrogen (>90%) are attained.
• The process can also achieve a relatively practical rate of about 50 mg N/(L·d).
• Regeneration of Fe(III) is important to save iron consumption for nitrogen removal.

## Graphical abstract

**Figure:**
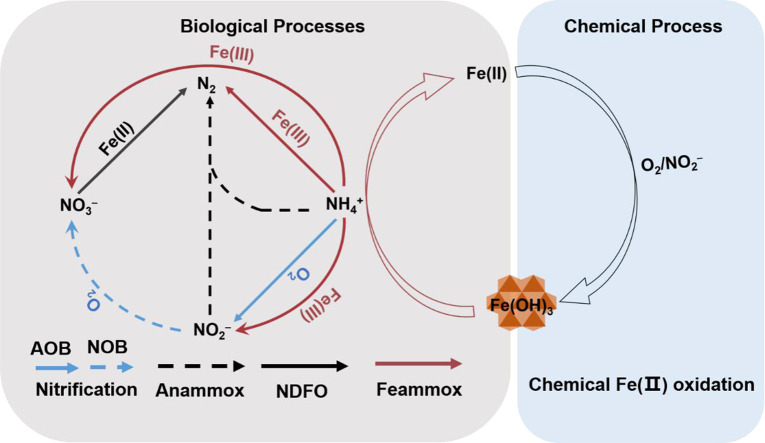

## 1 Introduction

Wastewater treatment plants are currently reliant on the conventional nitrification and denitrification process for nitrogen removal, which is not only energy-intensive but also overlooking a valuable opportunity to recover bioenergy from wastewater (Liu et al., 2019). The autotrophic nitrogen removal process, which does not require organic carbon for nitrogen removal, is therefore considered essential in next-generation wastewater treatment processes (Kartal et al., 2010). In light of this, extensive studies have been conducted on the autotrophic nitrogen removal pathway via partial nitritation and anammox (PN/A) (Gao et al., 2023; Zheng et al., 2023). Nonetheless, the robustness of the PN/A process remains a challenge under the conditions of low influent ammonium concentration and ambient temperature (Cao et al., 2017; Wang et al., 2021; Li et al., 2021; Meng et al., 2022; Su et al., 2023). Consequently, there is a need to seek alternative options for autotrophic nitrogen removal (Pang et al., 2022; Wang et al., 2022).

Fe(III) reduction coupled to anaerobic ammonium oxidation (Feammox) is an important pathway in the geochemical cycles of nitrogen and iron in natural environments (Yang et al., 2012). This process has been monitored across diverse ecosystems, such as in eutrophic lake (Yao et al., 2019), freshwater sediment (Z. Yao et al., 2020), mangrove soil (Guan et al., 2018), farmland ecosystems (Ding et al., 2020), and intertidal wetland (Li et al., 2015). In the Feammox process, ammonium can be converted to nitrogen gas (N_2_) (Li et al., 2019; Ma et al., 2021), nitrite (Huang et al., 2015; Sawayama., 2006), and nitrate (Clément et al., 2005; Z. Yao et al., 2020) (Eqs. (1)‒(3)). Among them, N_2_ is a favourable product because Eq (1) can produce more energy thermodynamically. Indeed, N_2_ has been confirmed as the predominant product of Feammox in natural ecosystems, particularly at neutral pH levels (Li et al., 2019; Yang et al., 2012).

(1) 3Fe(OH)_3_ + 5H^+^ + NH_4_^+^ → 3Fe^2+^ + 9H_2_O + 0.5N_2_ ∆_r_G_m_ = ‒245 kJ/mol

(2) 6Fe(OH)_3_ + 10H^+^ + NH_4_^+^ → 6Fe^2+^ + 16H_2_O + NO_2_^‒^ ∆_r_G_m_ = ‒164 kJ/mol

(3) 8Fe(OH)_3_ + 14H^+^ + NH_4_^+^ → 8Fe^2+^ + 21H_2_O + NO_3_^‒^ ∆_r_G_m_ = ‒207 kJ/mol

In the context of wastewater treatment, Feammox presents itself as a viable avenue for autotrophic nitrogen removal and recent studies have revealed an important role of Feammox in diverse engineering systems (Cao et al., 2022; Li et al., 2021; Tan et al., 2022; Tian et al., 2020; Xia et al., 2022). For example, anaerobic ammonium removal was observed after adding ferric salts to anaerobically digested sludge (Y. Yang et al., 2018); the phenomenon of Feammox was observed in anammox reactors after long-term cultivation (Li et al., 2018; Yang et al., 2021); and a recent study also showed the feasibility of using Feammox contributing to nitrogen removal (Hu et al., 2022). Nevertheless, to the best of our knowledge, most of these studies focus on the observation of Feammox phenomenon in engineering systems, rather than harnessing this distinctive reaction to facilitate beneficial autotrophic nitrogen removal. Whether Feammox-based autotrophic nitrogen removal can achieve very high-level nitrogen removal remains to be investigated. Further, the application of Feammox for ammonium removal also faces an intrinsic challenge of the substantial iron consumption, indicated by the molar ratio Fe/NH_4_^+^ of 3:1 according to Eq. (1). This concern necessitates the regeneration of Fe(III) from Fe(II) to mitigate the excessive iron usage. Reactions such as nitrite/nitrate-dependent iron oxidation (NDFO) can facilitate the conversion of Fe(II) back to Fe(III) (Eqs. (4) and (5)) (Cheng et al., 2023; Yang et al., 2021). Alternatively, the regeneration of Fe(III) from the oxidation of Fe(II) can also be catalyzed by oxygen (Xu et al., 2024; Y. Yang et al., 2021). Whether these abiotic and biotic processes can contribute to in-situ Fe(III) regeneration, to substantially reduce chemical iron demand, is a critical barrier for process development.

(4) 10Fe^2+^ + 12H^+^ + 2NO_3_^−^ → 10Fe^3+^ + 6H_2_O + N_2_ 6Fe^2+^ + ∆_r_G_m_ = −457 kJ/mol

(5) 8H^+^ + 2NO_2_^−^ → 6Fe^3+^ + 4H_2_O + N_2_ ∆_r_G_m_ = −438 kJ/mol

The primary goal of this study is to examine the feasibility of Feammox-based reaction for high-level autotrophic nitrogen removal in wastewater. To this end, a laboratory-scale up-flow bioreactor was set up with Fe(OH)_3_ as an iron source. Over the 200-day operation, the nitrogen removal performance was monitored regularly. A series of ex-situ batch tests were also conducted to confirm key microbial and chemical reactions driving the anaerobic ammonium removal and the regeneration of Fe(III) from Fe(II). The potential functional microorganisms involved in this novel system were analyzed by 16S rRNA gene amplicon sequencing. These results thus add a new toolkit for autotrophic nitrogen removal in wastewater.

## 2 Results

### 2.1 Performance of Feammox-based nitrogen removal

**Fig. 1:**
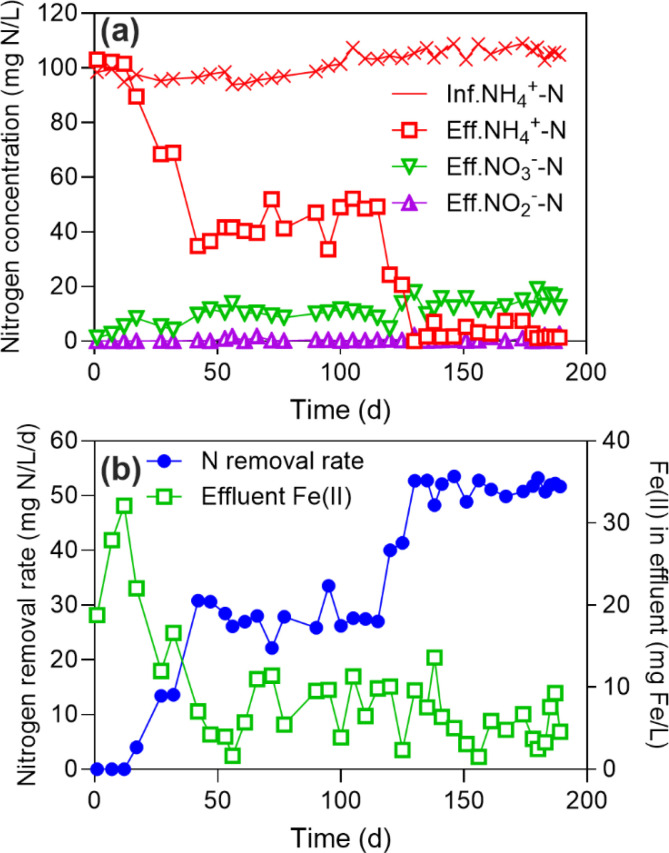
Long-term results of Fe(OH)_3_-added bioreactor: concentrations of NH_4_^+^-N, NO_2_^‒^-N, NO_3_^‒^-N in influent and effluent (a), ammonium nitrogen removal rate and Fe(II) concentration in the effluent (b).

The influent ammonium concentration was maintained at 100 mg N/L throughout the operation. An increase in effluent ammonium was observed in the first 16 days, which was likely induced by the decomposition of microorganisms in the seed anammox sludge. There was a dramatic decrease in effluent ammonium concentration after Day 17 (Fig. 1a), resulting in a significant increase in the removal efficiency of ammonium from 8.2% to 79.4% on Day 125. The effluent ammonium concentration continued to decrease down to 3.0 ± 2.4 mg N/L till the end of the bioreactor operation. This gave an ammonium removal rate of 51.5 ± 1.5 mg N/L/d (Fig. 1b), a high-level ammonium removal efficiency of 97.2 ± 2.3%, and a high-level total nitrogen (TN) removal efficiency of about 90%.

The effluent nitrite concentration was low throughout the experiment with an average value of only 0.5 ± 0.7 mg N/L (Fig. 1a). The concentration of nitrate in the effluent stabilized at around 10.0 mg N/L. The ratios of produced NO_3_^−^-N to the removed TN were determined to be less than 0.2, slightly higher than the theoretical value (i.e., 0.13) by the anammox reaction. This suggests the presence of other nitrate production pathways in addition to the anammox reaction, e.g., the Feammox reaction (Eq. (3)).

The pH in the influent was relatively stable (6.72‒7.17). The pH in the bioreactor slightly increased, but the difference between them was significant (*p* < 0.05) in the last 30 days of operation (Fig. S1). Indeed, Feammox-based nitrogen conversion is a proton-consuming process (Hao et al., 2024).

The concentration of dissolved Fe(II) in the effluent stabilized in the range of 1.5‒10.0 mg/L after Day 40, indicating a reduction from Fe(OH)_3_ to Fe(II) in the bioreactor. However, the measured Fe(II) concentration is much lower than the theoretical Fe(II) value, calculated as in the range of 265.2‒1659.5 mg/L according to the total electron balance in Feammox (Text S1). One possible reason might be that Fe(II) produced by the Feammox reaction could be re-oxidized to Fe(III) by electron acceptors, such as nitrate via the NDFO pathway. Another reason might be that the formation of insoluble ferrous precipitation, such as Fe_3_(PO_4_)_2_·7H_2_O [solubility product constant (*K_sp_*) = 10^−40.74^] (Liu et al., 2018) and FeCO_3_ [*K_sp_* = 10^−10.59^] (Rumble., 2017), could prevent the loss of Fe(II) in the effluent.

### 2.2 Increased Feammox-based nitrogen conversion rate identified by *ex situ* batch tests

**Fig. 2:**
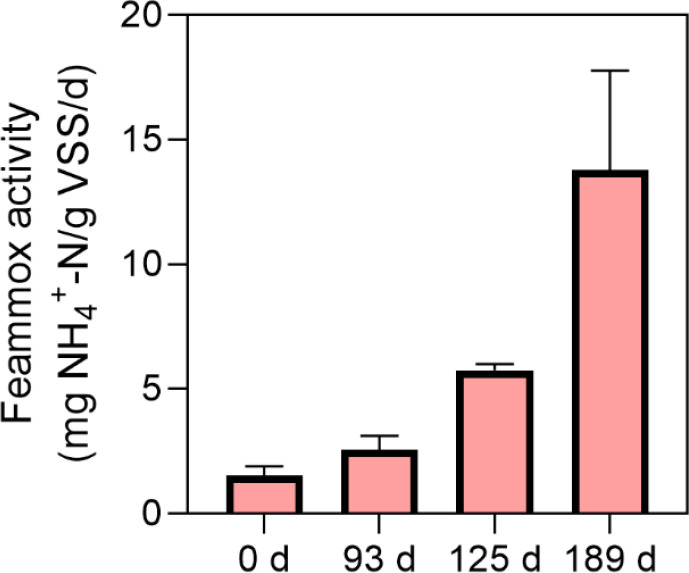
Feammox-based nitrogen conversion rate measured by *ex situ* batch tests for the sludge collected from the bioreactor on Days 0, 93, 125, and 184. Error bars represent standard deviations.

Feammox-based nitrogen conversion rate was examined by *ex situ* batch tests along with the long-term bioreactor operation (Fig. 2). Simultaneous ammonium consumption and Fe(II) production were observed in these tests with the sludge taken from the bioreactor on Days 0, 93, 125, and 184. No nitrite or nitrate accumulated during the tests, suggesting that the Feammox reaction likely converted ammonium directly to nitrogen gas (Eq. (1)). Notably, the measured Feammox rate gradually increased from a very low level of 1.5 mg N/(g volatile suspended solids (VSS)·d) on Day 0 to 5.8 mg N/(g VSS·d) on Day 125, and finally reached 13.8 mg N/(g VSS·d) on Day 189. The substantial rate increase indicated the enrichment of Feammox-related microorganisms along with long-term bioreactor operation.

### 2.3 Biological and chemical NDFO activities identified by *ex situ* batch tests

To provide evidence for the presence of NDFO reactions, *ex situ* batch tests were performed with the sludge collected from the bioreactor on Day 184. In the first abiotic group with the addition of nitrate and Fe(II), there was no significant variation in nitrate and Fe(II) within 25 days in the abiotic batch tests (*p* > 0.05) (Fig. S2a), suggesting the limited chemical reaction rate. In the corresponding biotic group, the concentration of Fe(II) gradually decreased from 271.6 mg/L to 127.3 mg/L in 25 days, and nitrate decreased from 43.9 mg N/L to 28.9 mg N/L without ammonium or nitrite accumulation (Fig. S2a). This result was in line with previous studies using ferrous to biologically reduce nitrate in polluted groundwater (Margalef-Marti et al., 2020), suggesting the presence of biological nitrate reduction driven by Fe(II) oxidation (Eq. (4)).

**Fig. 3:**
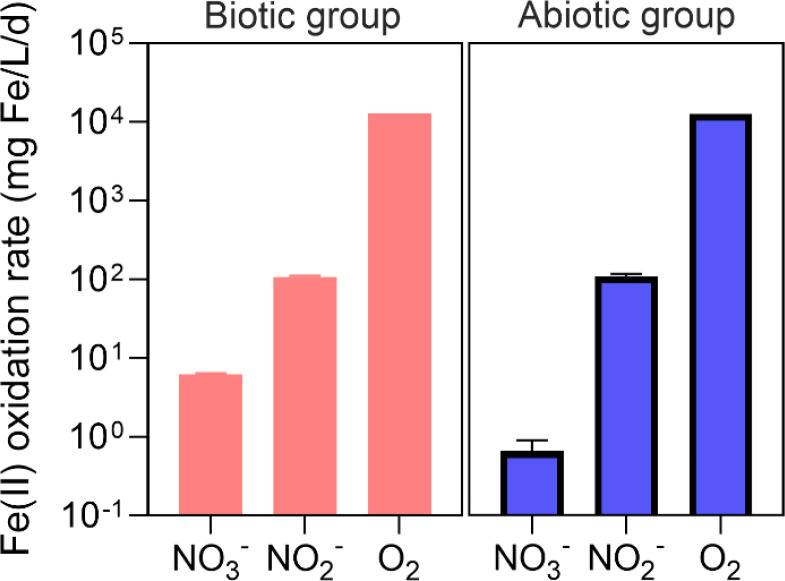
Measured rates of Fe(II) oxidation with different oxidants (O_2_, NO_2_^−^, and NO_3_^−^) in abiotic and biotic tests. The sludge used in the biotic tests was collected from the bioreactor on Day 184. Error bars represent standard deviations.

The second group was added with nitrite and Fe(II). Notably, Fe(II) significantly decreased from 214.8 mg/L to 4.1 mg/L within 24 h while nitrite decreased from 24.6 mg N/L to 5.8 mg N/L in the abiotic tests (Fig. S2b). The chemical oxidation rate of Fe(II) was determined to be 210.7 mg/(L·d) (Fig. 3), and the stoichiometric ratio of consumed Fe(II) and consumed nitrite (ΔFe(II)/ΔN) was 2.8, very close to the theoretical ratio of NDFO (Eq. (5)). The phenomenon was also observed in biotic tests with the addition of Feammox sludge. The Fe(II) decreased at a rate of 161.6 mg/(L·d) and the ΔFe(II)/ΔN ratio was determined to be 3.0 (Fig. S2b). These similar results from abiotic and biotic tests suggest that the chemical oxidation of Fe(II) by nitrite likely dominated the biological reaction in the bioreactor.

Oxygen was not supplied to the bioreactor intentionally, but dissolved oxygen was still detectable at a level of ∼0.05 mg O_2_/L due to mixing. As such, the oxidation of Fe(II) by O_2_ was also considered (Fig. S2c). The rates obtained from biotic and abiotic groups were found to be similar (Fig. 3). However, the Fe(II) oxidation rate by oxygen was two to four orders of magnitude higher than that driven by nitrate and nitrite. Indeed, oxygen has a very high redox potential compared to nitrite and nitrate (Melton et al., 2014). Together, these results suggest that Fe(II) oxidation in the bioreactor was driven by chemical reactions with oxygen and nitrite, compared to orders of magnitude slower biological Fe(II) oxidation rates using oxygen, nitrite, and nitrate.

### 2.4 Microbial community analysis

Relative abundances of bacteria at the genus level in three collected samples are presented in Fig. S3. The sequencing results revealed that the relative abundance of anammox *Candidatus* Brocadia was 23.0% in the seed sludge and decreased to only 1.6% on Day 187. This suggests that anammox might not be the primary contributor to the achieved high-level nitrogen removal at the end of the bioreactor operation.

Microorganisms performing the Feammox reactor have not been well understood. A strain *Acidimicrobiaceae* A6 was the only reported ammonium-oxidizing iron reducer (Huang et al., 2018), which was not found in the present study. Previous studies suggested that dissimilatory iron reduction bacteria (DIRB) may participate in the anaerobic ammonium oxidation in Feammox (Li et al., 2015). In this study, the total relative abundances of typical DIRB, such as *Geobacter*, Geobacteraceae_unclassified, Geobacteraceae_uncultured and *Geothrix* (Ding et al., 2014; Esther et al., 2015), increased from < 0.01% in the seed sludge to 0.6% on Day 187. *Ignavibacterium* was reported to use iron compounds as an electron acceptor (Choi et al., 2022; Stern et al., 2018), and its abundance increased from 0.2% in the seed to 2.1% on Day 187. The relative abundance of *Paludibaculum*, reported to use amorphous Fe(OH)_3_ as an electron acceptor (Kulichevskaya et al., 2014), also increased from 0.02% to 0.55% from Day 0 to Day 187. Along with the achievement of high-level nitrogen removal performance, the most dominant genus was PHOS-HE36, with a relative abundance of 18.7%, which is however still unclassified.

Abundances of nitrifying bacteria dramatically decreased in the bioreactor, as no oxygen was provided intentionally. Specifically, the relative abundance of ammonia-oxidizing *Nitrosomonas* genus decreased from 1.5% in the inoculum to 0.5% on Day 187, and the nitrite-oxidizing *Nitrospira* genus decreased from 5.0% to 1.8%. With the significantly decreased abundance, the contribution of nitrifying bacteria to nitrogen conversion should be neglectable. As confirmed by *ex situ* batch tests (Fig. S4), the maximal aerobic ammonia- and nitrite-oxidizing activities of the Feammox sludge were only 2.6 − 3.6 mg NH_4_^+^-N/(L·d) and 2.9 − 3.4 mg NO_2_^-^-N/(L·d), respectively.

## 3 Discussion

In the present study, high-level ammonium (∼97%) and TN (∼90%) removal was achieved in a Fe(OH)_3_-added bioreactor without any active oxygen supply. Compared with previous studies operating Feammox systems in continuous mode with similar influent ammonium and operational conditions, this study presented very high nitrogen removal efficiency, along with a practically useful removal rate (Table 1).

**Table 1:** Profiles of Feammox-based nitrogen removal in the literature and this study. DFAR: demand of Fe(Ⅲ) to ammonium nitrogen removed.

Seed sludge	Suspended solid (mg/L)	HRT (h)	Temp ( °C)	Influent NH_4_^+^-N (mg/L)	Removal efficiency of NH_4_^+^-N (%)	Removal rate of NH_4_^+^-N (mg/L/d)	Fe(Ⅲ) compound	DFAR	Note	Reference
Activated sludge	–	24	20 ± 2	32.5	99	32.5	–	–	DO of 0.58 mg/L, C/N ratio of 7.2	Ma et al., 2022
Activated sludge	9900*	24	35	40‒60	97.9‒99.5	40‒60	Fe_2_O_3_ + Fe	1.30	Activated carbon of 50 g/L	Cao et al., 2022
Anammox sludge	10,000	24	32 ± 2	50	68	34.0	FeCl_3_	0.27	Dosage of NO_3_^-^	Li et al., 2018
Fe-rich anaerobic sludge	–	144	30 ± 2	50	> 91	7.58‒8.33	Ferrihydrite	1.12	C/N ratio of 1.4	Le et al., 2021
Anaerobic sludge	2000	48	–	100	53	26.5	Fe_2_O_3_	0.54	N/A	Zhu et al., 2022
Anammox sludge	2668 (904*)	48	23 ± 3	100	52.3	26.2	Fe_2_O_3_	0.17	N/A	Hu et al., 2022
Anammox granular sludge	–	48	32 ± 2	100	80	40	FeCl_3_	0.29	N/A	Li et al., 2018
Fe-rich sludge	–	144	30‒33	100	99	16.5	Ferrihydrite	2.5	C/N ratio of 2.5	Nguyen et al., 2023
–	–	14	25	15	90–92	0.96–0.98	Fe_3_O_4_	–	Carrier	Liang et al., 2023
Activated sludge	2000*	48⁎⁎	25–30	36.3–52.9	100	–	Fe_2_O_3_	–	SBR⁎⁎⁎	Hao et al., 2024
Feammox sludge	5000	0.1	22	195–200	43.4	20,832	FeCl_3_	6.5	SBR	González et al., 2024
Anammox sludge	2900	48	23 ± 3	100	97.2 ± 2.3%	51.5 ± 1.5	Fe(OH)_3_	0.033	No carbon source	This study

⁎ Volatile suspended solid content (mg/L).

⁎⁎ Cycle time (h).

⁎⁎⁎ SBR: sequencing batch reactor.

Groups of batch tests were performed to shed light on potential mechanisms for the high-level autotrophic nitrogen removal. Firstly, there are three major reactions responsible for ammonium removal, namely Feammox, anammox, and aerobic ammonia oxidation. The limited aerobic ammonia oxidation activity (Fig. S4a), the substantially decreased abundance of anammox bacteria (Fig. S3), and the gradually enhanced Feammox activity (Fig. 2) jointly suggest that the Feammox reaction was primarily driving the enhanced ammonium removal in the present bioreactor. Furthermore, the product of ammonium oxidation by Feammox could be N_2_, nitrite, and nitrate (Eqs. (1)–(3)). Feammox(N_2_) has lower Gibbs free energy than Feammox(NO_3_^‒^) and Feammox(NO_2_^‒^), indicating N_2_ is a more thermodynamically favourable product. Indeed, a previous study showed that nitrite and nitrate were produced below pH 6.5 by the Feammox reaction while N_2_ was yielded over a wide pH range (Yang et al., 2012). Ding et al. (2014) also evaluated the contribution of three pathways of Feammox to the nitrogen loss in paddy soils, which showed that 67–78% of nitrogen loss was converted to nitrogen gas by Feammox. Together with the observed low levels of nitrite and nitrate in the effluent, it is reasonable to assume the majority of ammonium was removed as nitrogen gas by the Feammox reaction.

In terms of nitrite consumption, there are four potential pathways, namely aerobic nitrite oxidation, anammox, and biological and chemical NDFO reactions (Eq. (5)). Again, the limited aerobic nitrite oxidation activity (Fig. S4b) and the substantially decreased abundance of anammox bacteria (Fig. S3) suggest that they might not be competitive in utilizing nitrite compared to the other two reactions. Moreover, the chemical reaction between Fe(II) and nitrite was shown to dominate the biological counterpart, highlighting that nitrite was likely primarily removed via the chemical pathway. In contrast, the nitrate was mainly removed from the biological NDFO reaction (Eq. (4)), while the consumption through the chemical reaction was limited.

Compared with denitrification processes, the present nitrogen removal by Feammox does not require organic carbon sources. This emphasizes the feasibility of further integrating this new process with upfront organics capturing to maximize bioenergy recovery from wastewater – an important concept that has not been well achieved. In recent studies, researchers have attempted to combine PN/A with Chemically Enhanced Primary Treatment (CEPT) to achieve this goal (Hu et al., 2023). Compared to this previous report, the present study indicates that anaerobic Feammox can be used instead of the PN/A process, thereby saving additional energy for aeration. Furthermore, the demand of Fe(III) for ammonium removal (DFAR) was determined to be only about 0.033 in the whole period of the bioreactor operation, suggesting its chemical consumption is also super low compared to continuous iron dosing in the CEPT process.

## 4 Conclusions

This study demonstrated that the Feammox bioreactor successfully achieved high ammonium efficiency (>97% on average). Without the addition of organic carbon, the total nitrogen removal efficiency reached approximately 90% at a relatively practical rate of ∼50 mg N/(L·d). Regeneration of Fe(III) from Fe(II) occurred in the system, likely driven by microbial nitrate reduction and chemical oxygen reduction. The regeneration of Fe(III) is critical to saving iron consumption in the Feammox-based nitrogen conversion process and supporting further development of this innovative process for nitrogen removal from wastewater.

## 5 Materials and methods

### 5.1 Reactor setup and operation

The sealed up-flow bioreactor with a total volume of 1.2 L was established (Fig. S5). The reaction zone was equipped with a pH probe (E201, Leici, China), and a magnetic stirrer (RCT B S025, IKA, Germany) was used to mix the solution and sludge in the bioreactor. Influent was introduced at the bottom of the bioreactor by a peristaltic pump (BT100–2 J, Longer Pump, China) while effluent was discharged from the top. A three-phase separator was employed in the reactor to improve sludge settlement and gas collection in a gas bag. The amount of wastewater treated by the reactor was approximately 0.76 L per day. Granular anammox sludge was obtained from a pilot-scale anammox reactor treating high-strength wastewater (Jiangsu, China). The pilot-scale anammox reactor has been operated for more than 3 years and the nitrogen removal efficiency is above 90%. The bioreactor was inoculated with the granular anammox sludge and the initial total suspended solids (TSS) was approximately 2.9 g/L after inoculation.

Fe(OH)_3_ used in this study was prepared by adding FeCl_3_ to deionized water and elevating the pH to 7.0‒7.3 by adding 1 M NaOH solution. The solution was centrifuged at 4000 rpm for 10 min (TDL-40B, Anting, China) to obtain the precipitates. The Fe(OH)_3_ prepared was added into the reactor on Day 0, 77, and 126, respectively, with the same dosage of 5 mM. Accordingly, the operation period of the bioreactor was divided into three phases (phase I (Days 0–76), phase II (Days 77–125), and phase III (Days 126–190)). Synthetic influent without organics was prepared including 383 mg/L NH_4_Cl, 200 mg/L MgCl_2_·6H_2_O, 136 mg/L CaCl_2_·2H_2_O, 27 mg/L KH_2_PO_4_, 500 mg/L NaHCO_3_, and 1 mL/L micronutrient solution (5000 mg/L EDTA, 430 mg/L ZnSO_4_·H_2_O, 240 mg/L CoCl_2_·6H_2_O, 250 mg/L CuSO_4_·5H_2_O, 220 mg/L NaMoO_4_·2H_2_O, 190 mg/L NiCl_2_·6H_2_O, 210 mg/L NaSeO_4_·10H_2_O, and 14 mg/L H_3_BO_4_). The corresponding influent ammonium concentration was approximately 100 mg N/L. The synthetic wastewater was flushed by the gas mixture N_2_/CO_2_ (80%/20%) for 30 min to remove oxygen. A gas bag filled with nitrogen gas was connected to the feeding tank to maintain the anaerobic environment and balance pressure. The reactor was operated at room temperature (23 ± 3 °C) and neutral pH with a fixed hydraulic retention time (HRT) of 2 days. Influent samples were taken from the feeding tank while the effluent samples were taken from the outlet of the bioreactor. Generally, the sampling time interval was 5 days, while between Day 178 and Day 189, more frequent sampling was conducted with the interval of 2 days. The pH and concentrations of ammonium, nitrite, nitrate and Fe(II) of the samples were analyzed. No oxygen was provided to the system, while dissolved oxygen concentration was measured as around 0.05 mg O_2_/L, indicating the presence of passive oxygen diffusion into the system.

### 5.2 Batch tests

Three series of batch tests were performed to examine different reactions in the present system (Table 2). Batch test 1 was carried out to evaluate the Feammox reaction. Briefly, sludge (30 mL) was taken from the bottom of the continuous bioreactor on Days 0, 93, 125, and 184, which was washed with deionized water three times prior to the experiment. The washed sludge was transferred into serum bottles (100 mL) and the oxygen was removed by flushing N_2_/CO_2_ (80%/20%) for 30 min. The bottle was sealed tightly with tinfoil, and filled with a solution with similar composition as the synthetic influent for the continuous operating bioreactor. The working volume was 90 mL of each bottle. The initial concentrations of ammonium and Fe(OH)_3_ were 50 mg N/L and 535 mg/L, respectively. Liquid samples were taken from the serum bottles at 5-day intervals and the concentration of ammonium in the samples was analyzed.

**Table 2:** Overview of the batch tests.

Batch test	Condition	Duration	Aim
1	NH_4_^+^ + Fe(OH)_3_	25 d	To confirm the Feammox reaction
2a	NO_3_^−^ + Fe(II)	25 d	To confirm chemical and biological Fe(II) oxidation processes
2b	NO_2_^−^ + Fe(II)	48 h	
2c	O_2_ + Fe(II)	32 min	
3	NH_4_^+^ + O_2_	36 h	To confirm the maximal rates of aerobic ammonia and nitrite oxidation
	NO_2_^−^ + O_2_	36 h	

A similar experimental design was applied to Batch tests 2 and 3. Batch test 2 aimed to investigate the chemical and biological Fe(II) oxidation rates using different electron acceptors. Moreover, abiotic tests were conducted without sludge as a control for Batch test 2, which also represented the chemical reactions in the proposed different conditions. The sampling time interval in batch test 2a was similar to that in batch test 1. Differently, batch test 2b for 48 h with a sampling time interval of 8‒16 h, while Batch test 2c lasted for 32 min with a sampling time interval of 1‒3 min. Concentrations of nitrogen (ammonium, nitrite and nitrate) and Fe(II) were measured in these samples. For Batch test 3, the serum bottles were flushed with air continuously during the experiment to measure the maximal activities of aerobic ammonia- and nitrite-oxidizing bacteria. Samples were taken at 6-h intervals and concentrations of ammonium, nitrite and nitrate were analyzed. All the batch tests were conducted in triplicate.

### 5.3 Analytical methods and calculations

The pH and dissolved oxygen were determined by the portable pH meter (Leici E‒301F, Shanghai INESA & Scientific Instrument, China) and dissolved oxygen meter (Oxi3310, WTW, Germany). Concentrations of ammonium, nitrite, and nitrate were examined using an ultraviolet spectrophotometer (DR 3900, HACH, USA) according to Standard Methods for the Examination of Water and Wastewater (APHA., 2015). Concentrations of Fe(II) in liquid samples were measured using the phenanthroline method, during which liquid samples were acidified to pH 2 and diluted with oxygen-removed deionized water. The absorbance at a wavelength of 510 nm was determined using an ultraviolet spectrophotometer (DR 3900, HACH, USA). To determine the concentration of total Fe, Fe(III) in the liquid samples was first reduced to Fe(II) using hydroxylamine hydrochloride, while Fe(II) was subsequently measured to represent the total Fe. The concentration of Fe(III) was calculated as the difference between total Fe and Fe(II). All liquid samples were filtered using 0.45 μm polyether sulphone membrane (TGMF60, Jinteng, China) before analysis.

Specific rate (mg N/(g VSS·d)) of aerobic ammonia oxidation, anammox, and Feammox was calculated as the slope of the decrease in the concentration of ammonium divided by the VSS concentration versus time. The specific rate (mg N/(g VSS·d)) of aerobic nitrite oxidation was calculated as the slope of the decrease in the concentration of nitrite divided by the VSS concentration versus time. The specific rate (mg N/(g VSS·d)) of NDFO was calculated as the slope of the decrease in the concentration of nitrate/nitrite divided by the VSS concentration versus time.

### 5.4 High-throughput sequencing and data analysis

The total genomic DNA of the sludge was extracted from 10 mL mixed liquor sample using E.Z.N.A. Soil DNA Kit (Omega Bio-Tek, Inc., US) according to the manufacturer's instructions. The purities and concentrations of DNA were determined with a Nanodrop UV spectrophotometer (Thermo Fisher Scientific, US). The V3-V4 regions of the 16S rRNA gene fragments were amplified from extracted DNA with primers 338F (5-ACTCCTACGGGAGGCAGCAG-3) and 806R (5-GGACTACHVGGGTWTCTAAT-3) through polymerase chain reaction (PCR) (Gene Amp 9700, ABI, USA). The PCR product was extracted from 2% agarose gel and purified using the AxyPrep DNA Gel Extraction Kit (Axygen Biosciences, Union City, CA, USA) according to manufacturer's instructions and quantified using Quantus™ Fluorometer (Promega, USA). Purified amplicons were pooled in equimolar and paired-end sequenced on an Illumina MiSeq PE300 platform (Illumina, San Diego, USA) according to the standard protocols by Majorbio Bio-Pharm Technology Co. Ltd. (Shanghai, China). More details of bioinformatics were described in the previous study (Li et al., 2023).

## CRediT authorship contribution statement

**Xiaohui Cheng:** Writing – original draft, Methodology, Investigation, Data curation. **Lanlan Hu:** Methodology, Investigation, Data curation. **Tao Liu:** Writing – review & editing, Conceptualization. **Xiang Cheng:** Supervision, Resources. **Jiyun Li:** Methodology, Investigation, Data curation. **Kangning Xu:** Writing – original draft, Supervision, Resources, Project administration, Formal analysis, Conceptualization. **Min Zheng:** Writing – review & editing, Visualization, Validation, Methodology, Conceptualization.

## Declaration of competing interest

The authors declare that they have no known competing financial interests or personal relationships that could have appeared to influence the work reported in this paper.

## Data Availability

Data will be made available on request

## Appendix Supplementary materials

Image, application 1
