# Supplementary material for: High-level nitrogen removal achieved by Feammox-based autotrophic nitrogen conversion
Source: Water Res X. 2024 Dec 3;27:100292. doi: 10.1016/j.wroa.2024.100292 (PMC11667699; doi:10.1016/j.wroa.2024.100292)
Supplement: Supplementary file 1 [file mmc1.docx]

**Supplementary Materials**

**High-level nitrogen removal achieved by Feammox-based autotrophic nitrogen conversion**

Xiaohui Cheng^1^, Lanlan Hu^1^, Tao Liu,^2^ Xiang Cheng^1^, Jiyun Li^3^,

Kangning Xu^1,*^, Min Zheng^4,*^

^1^Beijing Key Lab for Source Control Technology of Water Pollution, College of Environmental Science and Engineering, Beijing Forestry University, Beijing 100083, China

^2^Department of Civil and Environmental Engineering, The Hong Kong Polytechnic University, Hong Kong 999077, China

^3^School of Environment, Tsinghua University, Beijing 100084, China

^4^Water Research Centre, School of Civil and Environmental Engineering, University of New South Wales, Sydney, New South Wales 2052, Australia

***Corresponding authors**

Email address: [xukangning@bjfu.edu.cn](mailto:xukangning@bjfu.edu.cn) (Kangning Xu);

[min.zheng1@unsw.edu.au](mailto:min.zheng1@unsw.edu.au) (Min Zheng)

Number of pages: 5

Number of figures: 5

**Text S1. Calculation of the theoretical Fe(II) in effluent.**

Theoretical Fe(II) of Feammox process was calculated according to different products of the Equations 1‒3, as shown in following equations:

| (1)  (2)  (3)  The theoretical production of Fe(II) = (1) + (2) + (3) |
| --- |

**Text S2.** **Calculation of the Thermodynamic Favorability of Fe_2_O_3_-induced Feammox Reactions.**

The change in Gibbs free energy of Equation 1 was calculated to determine the thermodynamic feasibility of the Fe_2_O_3_-driven Feammox reactions using the following equation:

$$\text{aA}+\text{bB}\to\text{cC}+\text{dD}$$

$$\Delta G_{\text{r}}^{0} =\text{c}\Delta\text{G}_{\text{fC}}^{0} +\text{d}\Delta\text{G}_{\text{fD}}^{0} -\text{a}\Delta\text{G}_{\text{fA}}^{0} -\text{b}\Delta\text{G}_{\text{fB}}^{0}$$

R is the gas constant, which equals 0.008314 kJ mol^-1^ K. and T is the absolute temperature in Kelvin (287.15 *K*). Free energies of formation were obtained from Stumm and Morgan (1996): = ‒79.37 kJ mol^-1^, = ‒37.2 kJ mol^-1^, = ‒237.18 kJ mol^-1^, $\Delta\text{G}_{\text{r}}^{\text{0 }} (Fe^{2+} )$= ‒78.87 kJ mol^-1^, = ‒742.20 kJ mol^-1^.

1.5Fe_2_O_3_ + 5H^+^ + NH_4_^+^ → 3Fe^2+^ + 4.5H_2_O + 0.5N_2_

The chemical activity values used in the calculation are based on the Yang et al (2012): {NH_4_^+^} = 0.0002; {Fe_2_O_3_} = 1; {Fe^2+^} = 10^-12^, at a pH of 5. An activity of 1 was used for the solid phase hydrous Fe(Ⅲ) oxide minerals.

**Reference**

Stumm, W. and Morgan, J.J., 1996. Water chemistry. Encyclopedia of Environmental science, 3, 1142-1161.


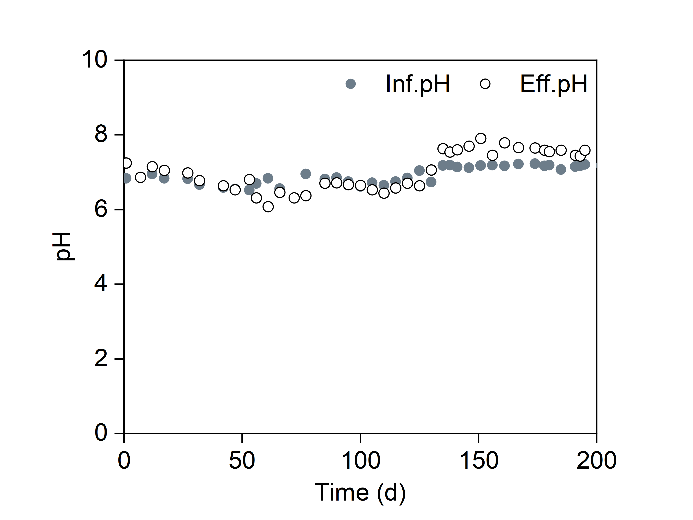


**Fig. S1.** pH of the influent and the effluent of the long-term Fe(OH)_3_-added bioreactor.

**Fig. S2.** Oxidation of Fe(Ⅱ) with different electron acceptors: (a) nitrate, (b) nitrite, and (c) oxygen. The biotic groups used sludge taken from the Feammox bioreactor on Day 184, while no sludge was added to the abiotic groups.


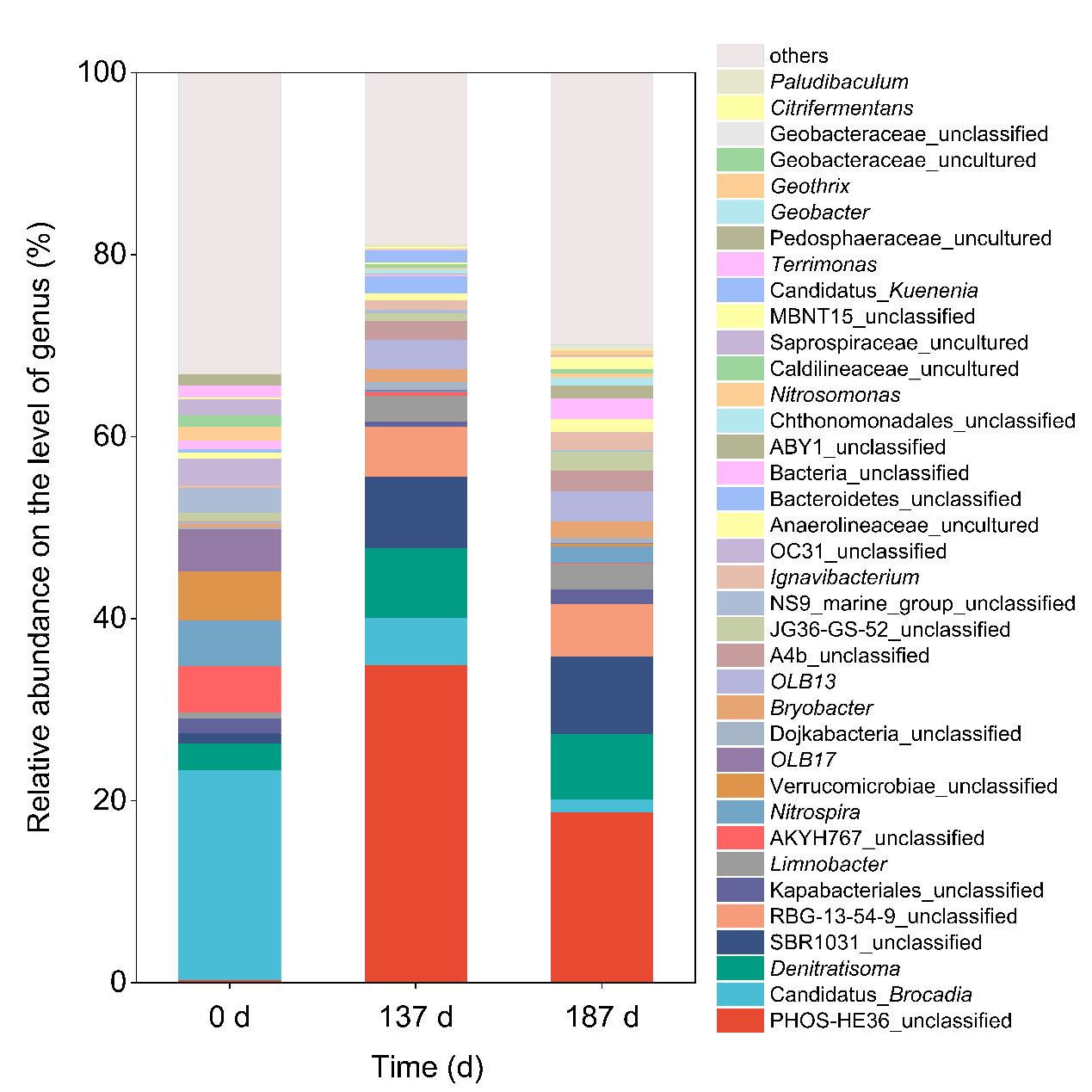


**Fig. S3.** Microbial community structure for biomass taken on Days 0, 137, and 187 was revealed by 16S rRNA gene amplicon sequencing. Most genera with relative abundance < 1% were collected as others.

**Fig. S4.** Activities of AOB (a) and NOB (b) were measured by aerating the biomass taken from the bioreactor on Day 184.


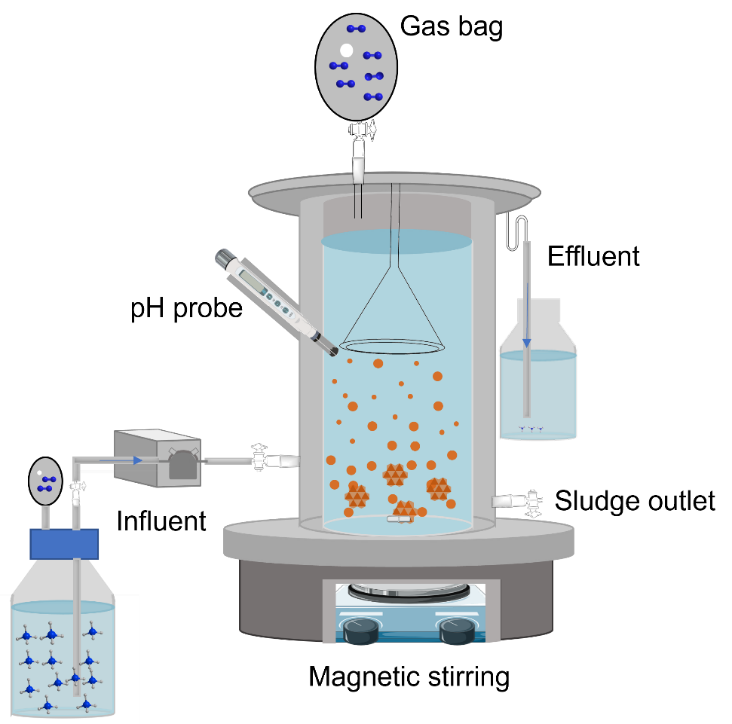


**Fig. S5.** Diagram of the up-flow Feammox bioreactor equipped with a three-phase separator.
